# Mosaic duplication of 8q24.1q24.3 detected by chromosomal microarray but not karyotyping in two unrelated fetuses with cardiac defects

**DOI:** 10.1186/s13039-021-00544-3

**Published:** 2021-05-18

**Authors:** Shaobin Lin, Shufang Huang, Xueling Ou, Heng Gu, Yonghua Wang, Ping Li, Yi Zhou

**Affiliations:** 1 Department of Obstetrics and Gynecology, The First Affiliated Hospital of Sun Yat-sen University, 58 Zhong Shan Er Road, Guangzhou, 510080 Guangdong Province China; 2 Prenatal Diagnosis Center, Department of Obstetrics and Gynecology, Guangdong Provincial People’ Hospital, Guangzhou, 510080 Guangdong Province China; 3 Faculty of Forensic Medicine, Zhongshan School of Medicine, Sun Yat-sen University, Guangzhou, 510080 Guangdong Province China; 4 NHC Key Laboratory of Male Reproduction and Genetics, Family Planning Research Institute of Guangdong Province, Guangzhou, 510080 Guangdong Province China

**Keywords:** 8q duplication, Fetus, Mosaicism, Congenital heart defect, Chromosomal microarray analysis

## Abstract

**Background:**

Discordance between traditional cytogenetic and molecular cytogenetic tests is rare but not uncommon. The explanation of discordance between two genetic methods is difficult but especially important for genetic counseling, particularly for prenatal genetic diagnosis.

**Case presentation:**

Two unrelated fetuses were diagnosed with cardiac defects by prenatal ultrasound examination, and invasive cordocentesis was performed to obtain cord blood samples for prenatal genetic diagnosis. For both fetuses, chromosomal microarray analysis (CMA) detected a novel approximately 27-Mb mosaic duplication with a high copy number of approximately six to seven copies on chromosome 8q24.1q24.3 that was not identified by karyotyping. To exclude artificial errors and validate laboratory detection results, multiple procedures including copy number variation sequencing, fluorescence in situ hybridization, and short tandem repeat and single-nucleotide polymorphism genotype comparison were performed, confirming the discordant results between CMA and karyotyping. The potential causes of discordance between CMA and karyotyping using fetal blood lymphocytes are discussed; we suggest that extrachromosomal DNA or cell-free DNA fragmentation originating from certain tumor tissues with 8q24.1q24.3 duplication might deserve further investigation.

**Conclusions:**

This study may be helpful for prenatal evaluation and genetic counseling for subsequent patients with similar mosaic 8q24.1q24.3 duplications. Additionally, more cases and further research are needed to understand whether mosaic 8q24.1q24.3 duplication is associated with certain genetic disorders and to investigate the causes of discordance between molecular and morphological methods.

## Background

Discordance between traditional cytogenetic and molecular cytogenetic tests is rare but not uncommon. Such differences are found mainly with regard to certain supernumerary chromosomes, such as isochromosomes and isodicentric chromosomes, or even some rare chromosomal trisomies. The most common types of chromosomal abnormalities reported to date include i(8p), i(9p), i(12p) and trisomy 9 mosaicism instead of partial monosomy/trisomy not containing a centromere [1]. Although the mechanism remains largely unexplained, it is largely attributed to the cell culture selection effect, tissue-restricted mosaicism or technical sensitivity [1–3]. The explanation of discordance between two genetic methods is difficult but especially important for genetic counseling, particularly for prenatal genetic diagnosis. Here, we report two unrelated fetuses with congenital heart defects (CHDs), in which an ~ 27-Mb mosaic duplication with a high copy number of approximately six to seven copies on chromosome 8q24.1q24.3 was detected by chromosomal microarray analysis (CMA) but not by traditional karyotyping of prenatal cord blood.

## Case presentation

### Case 1

**Fig. 1:**
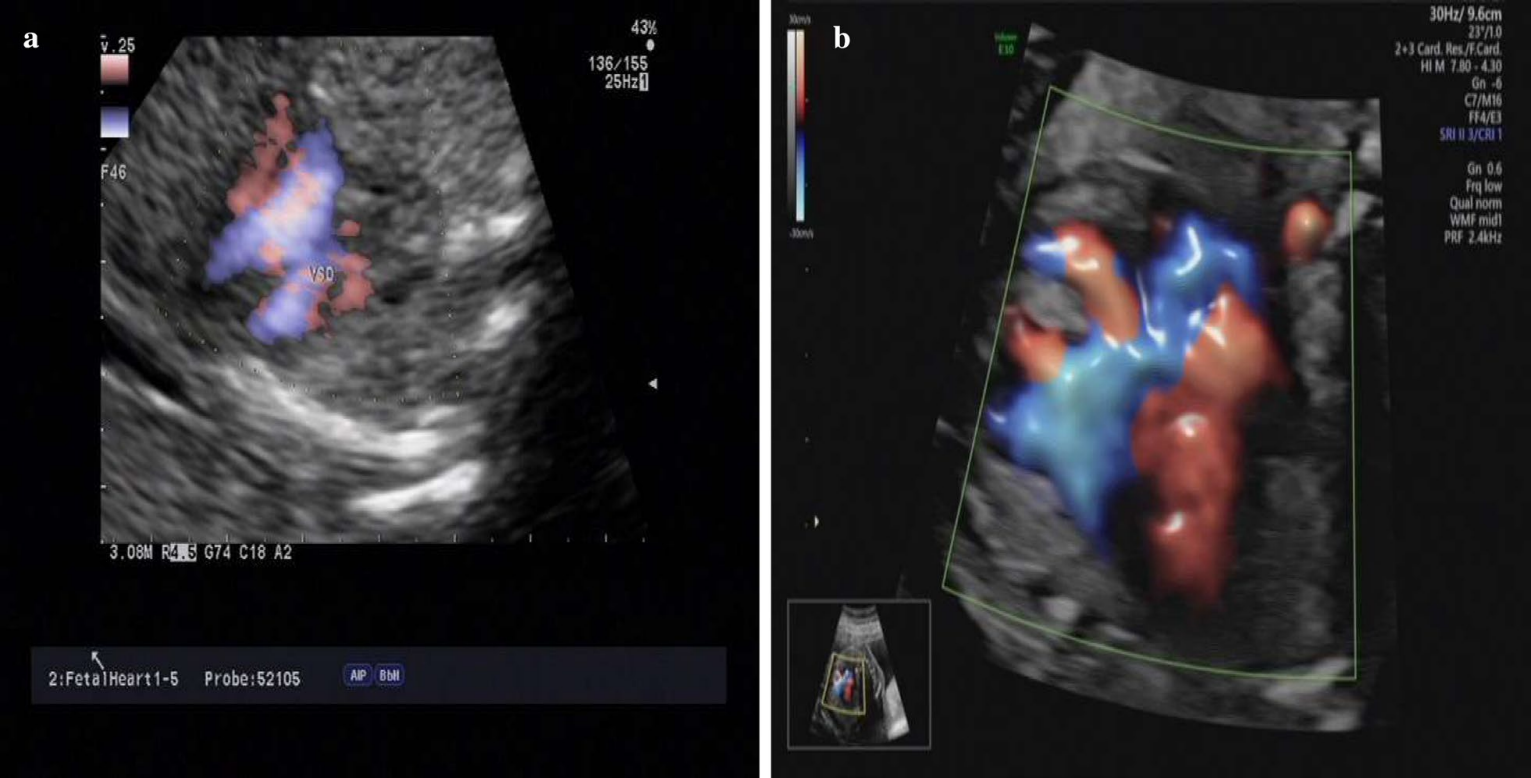
Ultrasound images of the fetuses. **a** An ultrasound image showed asymmetry of ventricular size with an enlarged right ventricle and a small left ventricle and ventricular septal defect in fetus 1 at 24 weeks of gestation. **b** An ultrasound image showed a complete atrioventricular septal defect in fetus 2 at 24 weeks of gestation

The mother in this case was 26 years old; gravida 2, para 0. The parents of the fetus were nonconsanguineous. The woman had a history of selective pregnancy termination of a fetus with tetralogy of Fallot. The current pregnancy was conceived naturally in 2018. Both maternal serum biochemical marker screening and noninvasive prenatal screening (NIPT) of fetal aneuploidy indicated low risk. At 18 weeks of gestation, ultrasound examination revealed a fetal echogenic bowel. Follow-up ultrasound examination suggested right fetal cardiac enlargement at 23 weeks of gestation. Further Doppler echocardiography at 24 weeks of gestation identified asymmetry of ventricular size with an enlarged right ventricle and a small left ventricle, ventricular septal defect of 4.6 mm, atrial septal defect of 7.8 mm and thin aortic arch (Fig. 1). The woman received genetic counseling and invasive cordocentesis sampling for karyotyping and CMA at 25 weeks of gestation.

### Case 2

The mother in this case was 33 years old; gravida 1, para 0. The couple was nonconsanguineous, and family history was unremarkable. The current pregnancy was conceived by intrauterine insemination in 2020. First-trimester maternal serum biochemical marker screening of fetal aneuploidy was missed, and the parents refused NIPT. Second-trimester serum screening of fetal aneuploidy showed a risk ratio of 1:905. At 24 weeks of gestation, ultrasound examination revealed a complete atrioventricular septal defect and severe tricuspid regurgitation in the fetus. At 27 weeks, follow-up ultrasound findings were complete atrioventricular septal defects, severe tricuspid regurgitation, pulmonary stenosis and ascites (Fig. 1). The woman received genetic counseling and invasive cordocentesis sampling for karyotyping and CMA at 27 weeks of gestation.

According to the decision of the parents, termination of pregnancy was performed in the two cases at 28 weeks and 29 weeks. The parents did not agree to further investigation using fetal tissues. The patients provided written informed consent for invasive prenatal examinations and for this study and publication of the data.

## Methods and results

**Fig. 2:**
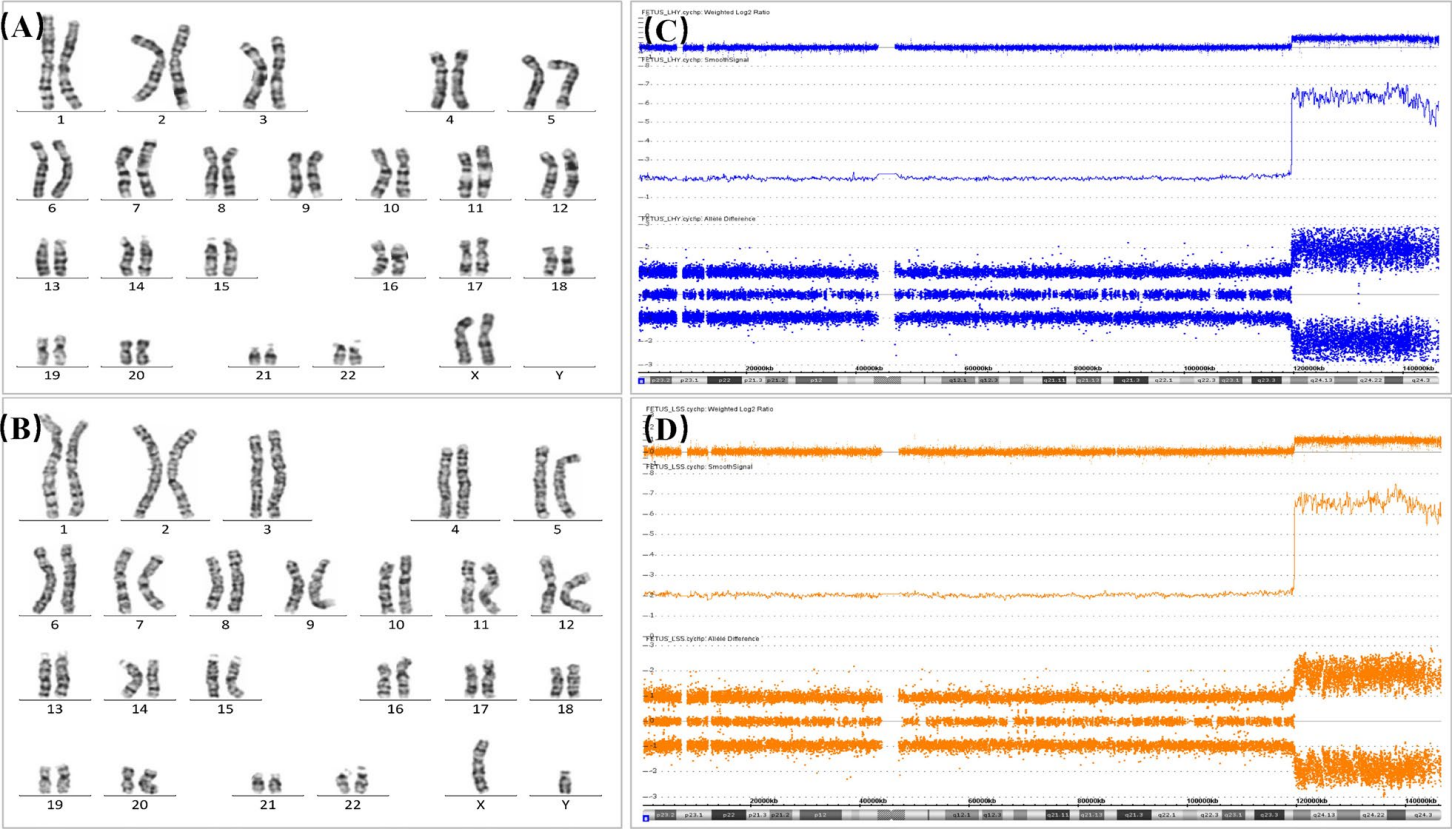
Karyotypes and CMA results. **a**, **b** Karyotyping of cord blood lymphocytes showed 46,XX and 46,XY in fetus 1 and fetus 2, respectively. **c**, **d** CMA performed on cord blood lymphocytes revealed approximately 27-Mb mosaic duplications with high copy numbers of approximately six to seven copies on chromosome 8q24.1q24.3 in fetus 1 and fetus 2, respectively

G-banded karyotyping was performed on peripheral blood and cord blood using standard procedures. Karyotyping of cord blood lymphocytes from the two fetuses showed normal karyotypes 46,XX and 46,XY (Fig. 2). The karyotypes of the parents were also normal.

However, CMA using CytoScan HD arrays with a single-nucleotide polymorphism (SNP) array platform (Thermo Fisher Scientific, Inc., Waltham, Massachusetts) performed using fetal blood lymphocytes detected mosaic duplications with a high copy number of approximately six to seven copies on chromosome 8q24.1q24.3. The 8q24 duplication segments in the two cases were almost unique, with a size of approximately 27 Mb: arr[GRCh37] 8q24.12q24.3(119328435_146295771) × 6 ~ 7 in case 1 and arr[GRCh37] 8q24.12q24.3(119261902_146295771) × 6 ~ 7 in case 2 (Fig. 2). The duplicated region ranged from 8q21.12 to 8q terminal (8qter), encompassing approximately 162 protein-coding genes, including the well-known oncogene *MYC*.

**Fig. 3:**
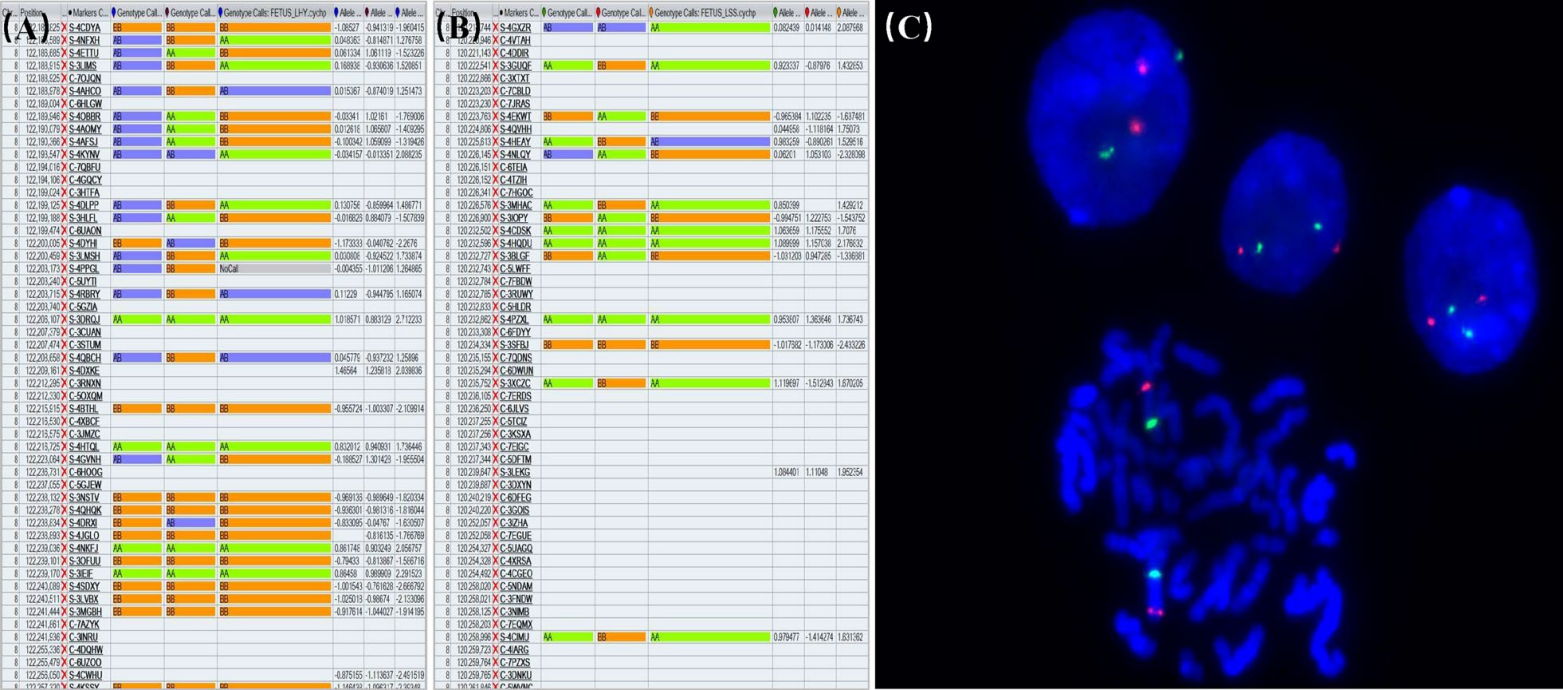
SNP-based Mendelian inheritance error (MIE) analysis for the fetus-parent trios and FISH image. **a**, **b** SNP genotype comparison showed that some SNP genotype calls in fetus 1 (**a**) and fetus 2 (**b**) did not match their mothers, indicating that they originated from the fathers. **c** FISH using a probe for the *MYC* (red fluorescence; green fluorescence CEN8 probe for the alpha satellite centromeric region of chromosome 8 as a control) located at 8q24 was performed on a cell suspension from cultured cord blood from fetus 2. The analysis showed a normal *MYC* gene copy number in both metaphase and interphase cells, indicating a nonduplicated 8q24 region

CMA performed on the parents of two fetuses did not reveal 8q24 duplications, indicating that the alterations were de novo duplications. According to SNP-based Mendelian inheritance error (MIE) analysis for the fetus-parent trios, the percentage of MIE on chromosome 8 was 2.16% for the mother in case 1 (MIE-mother markers/total markers: 838/38796) and 2.73% for the mother in case 2 (MIE-mother markers/total markers: 1058/38796), indicating that the 8q24 duplication originated from the father (Fig. 3). We further performed an SNP MIE analysis to investigate the relationship between the parental samples and fetal samples for the CMA test, which demonstrated the two samples to be related.

Then, next-generation sequencing for copy number variation detection using the NextSeq 500 platform (Illumina, San Diego, CA, USA) was performed on the two samples, as previously described [4], confirming the mosaic duplications in accordance with CMA (data are available upon request). Fluorescence in situ hybridization (FISH) using an *MYC* probe (red fluorescence; green fluorescence CEN8 probe for the alpha satellite centromeric region of chromosome 8 as a control) located at 8q24 was performed on a cell suspension of cultured fetal blood from case 2 (cell suspension not obtained in case 1). The *MYC* gene copy number was normal in both metaphase and interphase cells, indicating a nonduplicated 8q24 region (Fig. 3).

**Fig. 4:**
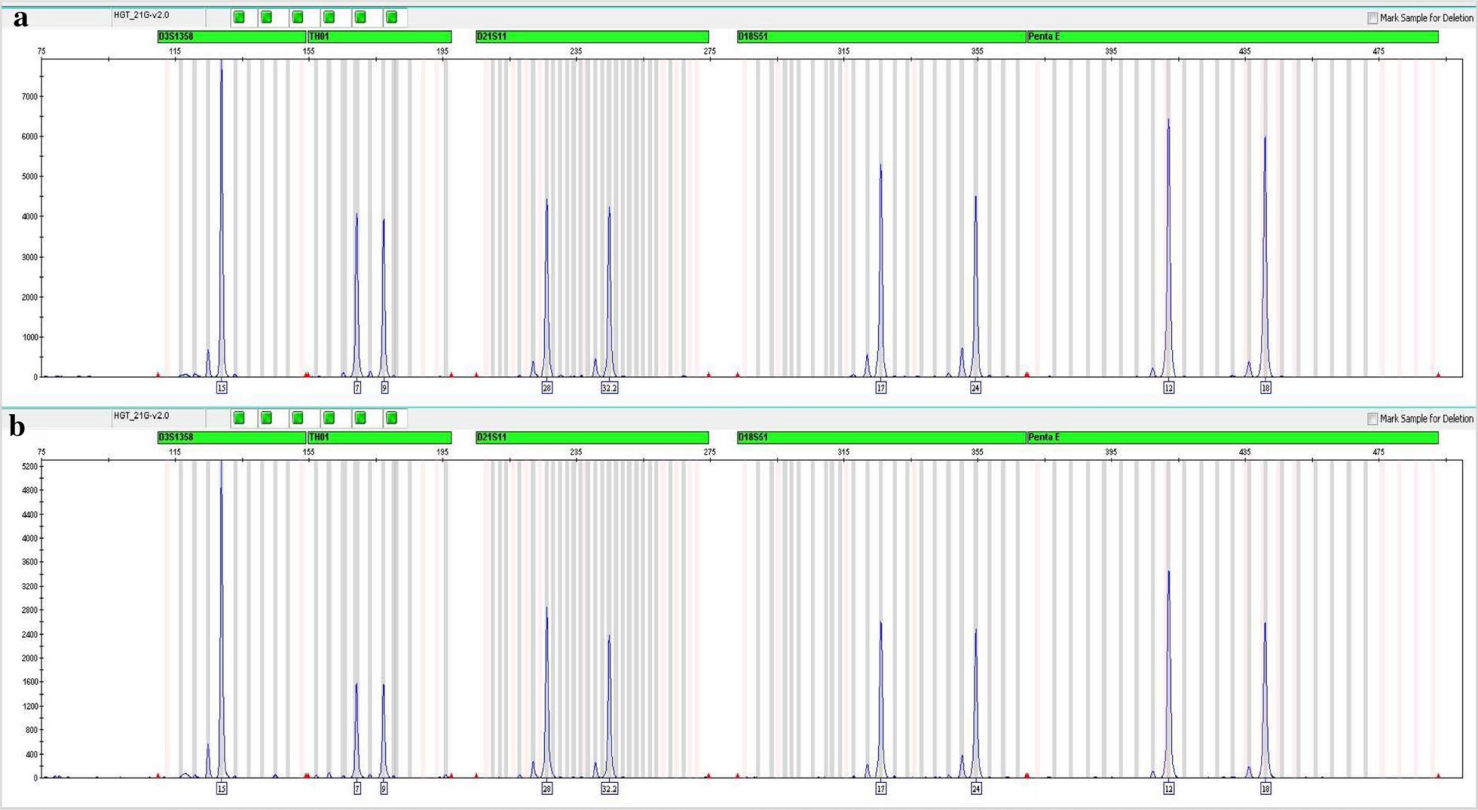
STR genotype results. A set of 21 polymorphic STR markers, including D3S1358, TH01, D21S11, D18S51, Penta E, D5S818, D13S317, D7S820, D16S539, CSF1PO, Penta D, vWA, D8S1179, TPOX, FGA, D19S433, D12S391, D6S1043, D2S1338, D1S1656 and Amelogenin, was used. STR genotype analysis using DNA from cells suspended in Carnoy's fixative fluid after karyotyping (**a**) and DNA from the CMA test (**b**) verified the concordance between the two samples in case 2 (five markers are shown in this image) and demonstrated that the samples indeed were from the same individual

Furthermore, we extracted genomic DNA from cells suspended in Carnoy's fixative fluid after karyotyping for case 2 (cell suspension not obtained in case 1) using the Chelex-100 method (Promega, Madison, WI, USA). Because the extracted DNA did not meet the quality requirement of CMA, we then performed short tandem repeat (STR) genotype analysis using it and DNA from the CMA test to determine concordance between the two samples. The result demonstrated that the samples indeed were from the same individual (STR markers and results shown in Fig. 4).

## Discussion and conclusions

In most cases, 8q duplication derives from chromosome 8 recombination or parental balanced translocations. In general, the characteristic features of cases with pure 8q duplications are not well defined because they often have various duplicated segments of 8q, carry other concomitant chromosomal rearrangements instead of pure 8q duplications, and show inconsistent phenotypic profile [5–7]. Pure terminal duplication of chromosome 8q24 is rare. To our knowledge, four cases with pure 8q duplication have been finely determined in previous studies [8–11]. These cases were reported to involve developmental disability, intellectual disability, epilepsy, CHDs and urinary defects, limb anomalies, cleft lip/palate, facial dysmorphism and feeding difficulties. Although a few similar features were observed among these cases, the exact critical region(s) or gene(s) has not yet been delineated. Moreover, CHDs were not consistently observed in these cases. Nevertheless, Digilio et al. [6] considered that CHDs, especially conotruncal heart anomalies, including tetralogy of Fallot, double-outlet right ventricle and malformation of the aortic arch, are a frequent finding in cases with 8q22-qter duplication. It should be noted that the case of 8q22-qter duplication in Concolino et al. [11] involved atrial and ventricular septal defects, in accordance with the cardiac anomalies observed in the present cases. The duplicated segments in the present cases ranging from 8q24.12 to 8qter suggest a narrow region associated with CHD. Regardless, more cases of discrepant duplicated segments of 8q would be valuable to further refine the critical region(s) or gene(s) associated with CHD.

Interestingly, the present two cases carried mosaic duplication of 8q with approximately six to seven copies, which has not been reported thus far. Surprisingly, mosaic duplications were detected by CMA but missed by karyotyping using fetal blood. To investigate artificial errors in the experimental procedure, we performed STR genotyping between the samples used for the CMA test and for karyotyping to confirm that the two sources of samples were from the same individual; we then performed SNP-based MIE analysis to verify that the fetal sample and parental samples had biological relationships. These verification tests excluded potential laboratory errors. The phenomenon of discordance between molecular techniques and traditional karyotyping is often observed in cases with Pallister-Killian syndrome, a tissue-limited mosaicism for the extra i(12p) chromosome [12]. Overall, i(12p) cells are more stable and show higher levels of fibroblasts, amniocytes and chorionic villus cells than found among rapidly growing blood lymphocytes, and they are difficult to detect in blood lymphocytes over time [3]. It is probable that i(12p) cells would be lost in the bone marrow and that they would also be replaced by rapidly growing normal cells stimulated by phytohemagglutinin in traditional karyotyping preparations [3, 12]. Nonetheless, these explanations do not appear to account for the causes of the mosaic 8q duplication detected by CMA and not karyotyping using fetal blood. Although both CMA and CNV-seq suggested mosaic 8q24.12q24.3 duplications in the two fetuses, we could not define the level of mosaicism because how the duplicated 8q24.12q24.3 segments were presented is still unknown without morphological evidence. Assuming that the duplicated 8q24.12q24.3 segments were present in a small supernumerary marker chromosome (sSMC) or a structural chromosomal rearrangement in cells, if the proportion of the cells was 1%, these cells would be expected to have approximately 600–700 × amplifications of the 8q24.12q24.3 segment, and if the proportion of the cells was 50%, these cells would be expected to have approximately 12–14 × amplifications of the 8q24.12q24.3 segment. In addition, the 8q24.12q24.3 region did not contain a centromere to constitute a stable sSMC, and the region was not found to have inserted or translocated to another chromosomal region to constitute a derivative chromosome. Therefore, an sSMC or a structural chromosomal rearrangement is not possible to detect in these cases, as it cannot be expected. Thus, we considered whether the duplicated segment had broken into numerous, undetected small fragments, a process called chromothripsis [13], which might insert into different genomic regions or be present in the form of extrachromosomal DNA (ecDNA) and recently proven to have an impact on tumor evolution and genetic heterogeneity. Accordingly, we used a probe for the *MYC* gene located at 8q24 to perform FISH on a cell suspension of cultured fetal blood lymphocytes after karyotyping in case 2 (cell suspension of case 1 could not be obtained), but the results showed a normal *MYC* gene copy number. This result appeared to exclude the possibility of insertion into different genomic regions due to chromothripsis, but it did not exclude the possibility of ecDNA. In fact, *MYC* or 8q24 amplification has been reported to be associated with an increased risk of some types of tumors in previous studies [14, 15]. Of note, several oncogenes encoded on ecDNA, including *MYC*, were the primary genes expressed in the cancer genomes [16]. Alternatively, another explanation for cytogenetically undetected 8q duplications is that they may be present as cell-free DNA (cfDNA) fragmentation originating from certain tumor tissues [17]. Therefore, the present mosaic 8q duplication might have consisted of numerous fragments of cfDNA released from specific tumor tissues. However, we did not uncover any potential clinical indication of a tumor in the fetuses or their parents based on prenatal examinations and parental medical records; and we also noted that fetal tumors with *c-MYC* or 8q24 amplification have not been reported in the literature. Whether 8q24 or *c-MYC* (or other genes located at 8q24) amplification was related to certain fetal tumors might deserve further investigation.

Notably, no specimens from either tumor patients or tumor cell lines were referred to our laboratory, in which prenatal cytogenetic and molecular genetic detections of rare human diseases in specimens from fetuses and their parents were mainly performed. Additionally, the time interval (one case in 2018 and another in 2020) between the present two fetuses referred to our laboratory was long; thus, it seems impossible that the duplicated genomic segments derived from a cell line contamination. Furthermore, SNP MIE analysis between the fetuses and parents also excluded the possibility of exogenous contamination.

The current study has certain limitations. For example, other types of cell lines, such as amniocytes, chorionic villi or skin fibroblasts, were not obtained from the two cases, and uncultured fetal blood lymphocytes were not reserved for further research. Furthermore, the FISH performed on cultured fetal blood lymphocytes could not exclude the potential artificial effects of cultured cells, which might induce a selective growth advantage or disadvantage for certain cell lines [18]. These unresolved issues may be important for further determining whether the mosaic 8q duplications were present in a tissue-limited manner and whether the duplications were lost during karyotyping preparations.

In brief, we present a novel mosaic 8q duplication with a copy number ranging from six to seven detected by CMA but not by karyotyping in two unrelated fetuses with CHDs. Through multiple procedures to exclude artificial errors and validate laboratory detection results, we were able to confirm the discordant results between CMA and karyotyping. This study may be helpful for prenatal evaluation and genetic counseling for subsequent cases with similar mosaic 8q duplications. In addition, more cases and further research are needed to understand whether mosaic 8q duplication is associated with certain chromosomal disorders and to investigate the causes of discordance between molecular and morphological methods.

## Data Availability

The datasets used and/or analyzed during the current study are available from the corresponding author on reasonable request.

## Abbreviations

cfDNA: Cell-free DNA
CHD: Congenital heart defect
CMA: Chromosomal microarray analysis
ecDNA: Extrachromosomal DNA
FISH: Fluorescence in situ hybridization
MIE: Mendelian inheritance error
NIPT: Noninvasive prenatal screening
SNP: Single nucleotide polymorphism
STR: Short tandem repeat
